# Clinical presentation, diagnosis, treatment, and outcome in 8 dogs and 2 cats with global hypoxic‐ischemic brain injury (2010‐2022)

**DOI:** 10.1111/jvim.16790

**Published:** 2023-06-14

**Authors:** Abbe Harper Crawford, Elsa Beltran, Cecilia‐Gabriella Danciu, Dylan Yaffy

**Affiliations:** ^1^ Clinical Science and Services Royal Veterinary College, Hawkshead Lane, North Mymms Hatfield AL9 7TA United Kingdom; ^2^ Pathobiology and Population Sciences Royal Veterinary College, Hawkshead Lane, North Mymms Hatfield AL9 7TA United Kingdom

**Keywords:** canine, cerebral, feline, hypoxia, ischemic

## Abstract

**Background:**

Global hypoxic‐ischemic brain injury (GHIBI) results in variable degrees of neurological dysfunction. Limited data exists to guide prognostication on likelihood of functional recovery.

**Hypothesis:**

Prolonged duration of hypoxic‐ischemic insult and absence of neurological improvement in the first 72 hours are negative prognostic indicators.

**Animals:**

Ten clinical cases with GHIBI.

**Methods:**

Retrospective case series describing 8 dogs and 2 cats with GHIBI, including clinical signs, treatment, and outcome.

**Results:**

Six dogs and 2 cats experienced cardiopulmonary arrest or anesthetic complication in a veterinary hospital and were promptly resuscitated. Seven showed progressive neurological improvement within 72 hours of the hypoxic‐ischemic insult. Four fully recovered and 3 had residual neurological deficits. One dog presented comatose after resuscitation at the primary care practice. Magnetic resonance imaging confirmed diffuse cerebral cortical swelling and severe brainstem compression and the dog was euthanized.

Two dogs suffered out‐of‐hospital cardiopulmonary arrest, secondary to a road traffic accident in 1 and laryngeal obstruction in the other. The first dog was euthanized after MRI that identified diffuse cerebral cortical swelling with severe brainstem compression. In the other dog, spontaneous circulation was recovered after 22 minutes of cardiopulmonary resuscitation. However, the dog remained blind, disorientated, and ambulatory tetraparetic with vestibular ataxia and was euthanized 58 days after presentation. Histopathological examination of the brain confirmed severe diffuse cerebral and cerebellar cortical necrosis.

**Conclusions and Clinical Importance:**

Duration of hypoxic‐ischemic insult, diffuse brainstem involvement, MRI features, and rate of neurological recovery could provide indications of the likelihood of functional recovery after GHIBI.

**Abbreviations**
BSH: British short hair
CKCS: Cavalier King Charles spaniel
CT: computed tomography
EEG: electroencephalography
FLAIR: fluid attenuated inversion recovery
GHIBI: global hypoxic‐ischemic brain injury
MRI: magnetic resonance imaging
ROSC: return of spontaneous circulation
T2W: T2‐weighted
VFCA: ventricular fibrillation cardiac arrest

## 1 INTRODUCTION

The brain accounts for approximately 2% of total body mass in humans, yet it utilizes approximately 20% of total body glucose because of its inherently high metabolic requirements.^1^ Limited local glucose storage and minimal reserves of high‐energy phosphates creates a dependency on a consistent supply of glucose and oxygen.^2^, ^3^ If cerebral perfusion fails to meet the energy demands and waste product removal requirements of the brain, a state of ischemia develops. Irreversible neuronal injury can begin within minutes of the onset of ischemia,^4^ with adenosine triphosphate (ATP) depletion resulting in failure of Na^+^K^+^ ATPase activity, intracellular sodium and water influx, and neuronal depolarization. Secondary release of excitatory neurotransmitters, particularly glutamate, and inflammatory mediators contributes to progressive injury, along with mitochondrial dysfunction, lipid peroxidation and vascular injury. Increasing intracellular concentrations of calcium activate programmed cell death pathways.^5^, ^6^, ^7^

Global hypoxic‐ischemic brain injury (GHIBI) occurs secondary to a generalized reduction in cerebral perfusion, for example, caused by cardiac arrest, hypovolemic shock, or severe hypotension. The cerebral cortex, hippocampus, and Purkinje neurons of the cerebellum are particularly susceptible to ischemic injury.^6^, ^7^ In dogs and cats, GHIBI has been reported after general anesthesia,^2^, ^8^, ^9^ in association with severe bite injuries,^5^ with strangulation^10^ and after cardiopulmonary arrest with resuscitation.^11^ Additionally, the use of mouth gags during dental procedures has been associated with GHIBI in cats.^12^, ^13^ Onset of neurological deficits after a global hypoxic‐ischemic event can vary, with reports documenting immediate onset to delays of several days.^2^, ^5^, ^9^ Equally, the range and severity of neurological deficits reported in association with GHIBI is highly variable.^2^, ^5^, ^9^, ^11^

In people, outcome after GHIBI is often poor, particularly in patients with out‐of‐hospital cardiopulmonary arrest, for whom survival rates of 7%‐30% have been documented.^14^, ^15^, ^16^ Available literature on outcome in cats and dogs is currently limited but positive outcomes have been recorded, potentially reflecting species differences in underlying etiology. A common cause in people is primary cardiovascular disease leading to out‐of‐hospital cardiac arrest, whereas anesthesia‐associated cardiorespiratory complications appear to be a major cause in domestic species.^2^, ^8^, ^9^, ^12^ In people, initial neurological deficits can be severe and neurological outcome is difficult to conclusively predict during the first 24‐72 hours after resuscitation.^17^ A combination of serial neurological assessments, electrodiagnostic evaluation, advanced imaging, and serum biomarkers often is utilized to support prognostication in human medicine.^18^ Limited data exists in veterinary medicine to guide prognostication. In our retrospective case series, we describe 10 clinical cases of GHIBI, including presenting clinical signs, diagnosis, treatment, and outcome and consider potential clinical indicators of prognosis. We hypothesized that prolonged duration of hypoxic‐ischemic insult and minimal or no neurological improvement in the first 72 hours post hypoxic‐ischemic insult would be negative prognostic indicators.

## 2 METHODS

Digital medical records from January 1, 2010 to November 30, 2022 from the Royal Veterinary College were searched for the following terms: “global brain ischemi*,” “global ischemi*,” “return of spontaneous circulation AND neurolo*,” “ROSC AND neurolog*,” and “anesthetic accident.” This approach identified 52 cases. Cases then were assessed for presence of complete medical records and a history of new onset neurological deficits after the suspected or documented hypoxic‐ischemic event with serial neurological assessments performed by a veterinary neurologist or neurology resident‐in‐training. Cases were excluded if clinical records were incomplete or unavailable for review, if neurological deficits were not recorded post‐hypoxic‐ischemic event, or if there was evidence of pre‐existing intracranial or concurrent systemic disease that could cause the identified neurological signs.

Information retrieved from the medical records included signalment, cause and duration of the hypoxic‐ischemic event, duration and type of clinical signs, general physical examination findings, diagnostic investigations performed and the associated results, serial neurological examination findings, treatments administered, and duration of hospitalization. Follow‐up information was obtained from daily neurological examinations while hospitalized, neurological status at hospital discharge, re‐examination appointments at the referral hospital, as well as from referring veterinary surgeons' clinical records. Rate of neurological improvement was considered rapid when repeated or continual daily improvements were documented on neurological examinations during hospitalization, whereas improvement was considered slow when minimal to no improvements were documented during hospitalization but evidence of diminishing neurological deficits was identified on follow‐up assessment(s). Short‐term outcome was defined as survival or non‐survival at 72 hours after the GHIBI.^19^ Long‐term outcome was defined as the neurological status at the last re‐examination appointment. The long‐term outcome scale comprised 4 scores:

- 0: dead or euthanized because of the severity of neurological deficits.
- 1: poor recovery with severe persistent neurologic deficits (such as abnormal mental status, persistent disorientation, blindness).
- 2: good recovery with mild persistent neurologic deficits (such as mild ataxia, partial visual deficits).
- 3: excellent recovery: the dog or cat was considered normal.

Data were analyzed for normality using the Shapiro‐Wilk test and presented as median and range.

## 3 RESULTS

### 3.1 In‐hospital cardiopulmonary arrest

Six dogs and 2 cats presented after an in‐hospital documented or suspected cardiopulmonary arrest. The median age was 2.5 years (range, 0.5‐8 years). Three females were neutered, 3 males were neutered and 2 were intact. Cardiopulmonary arrest during general anesthesia was reported in 4 dogs (during routine castration, Cavalier King Charles spaniel [CKCS]; routine ovariohysterectomy, Labrador retriever; ovariohysterectomy to manage pyometra, Shih Tzu [Video S1]; and surgical management of a prolapsed nictitans gland, Bichon Frise). A small crossbreed dog experienced cardiopulmonary arrest secondary to hypovolemia and anemia caused by severe hemorrhagic gastroenteritis (Video S2). A spaniel crossbreed dog presented for severe lethargy after a walk on a hot day, collapsed on arrival, was noted to be tachycardic and then went into cardiopulmonary arrest. A British short hair (BSH) cat experienced cardiopulmonary arrest during investigation of hypertrophic cardiomyopathy and fulminant left‐sided heart failure. Cardiopulmonary resuscitation was promptly initiated in all cases (6 at the referring veterinary surgeon's hospital and 1 [BSH] at our referral hospital) and return of spontaneous circulation (ROSC) was reported within a median of 3 minutes (range, 1‐5 minutes). Finally, a Maine Coon cat was reported to have experienced very prolonged recovery after general anesthesia for a dental procedure (whether a dental gag had been used was not recorded in the clinical records).

Neurological examination findings at a median of 9 hours post‐hypoxic‐ischemic insult (range, 1‐36 hours) included moderate (3) to severe (3) obtundation, disorientation (2), stupor (1), coma (1), decerebellate rigidity posture (1), nonambulatory tetraparesis (6), cerebellar ataxia (1), intention tremors (1), absent menace response bilaterally (6), absent vision (5), decreased (2) to absent (1) response to nasal stimulation and decreased to absent gag reflex (2). Generalized seizures were observed in the CKCS, Labrador retriever and Maine Coon cat. The spaniel crossbreed dog that collapsed after a walk was transferred to our hospital intubated and manually ventilated. Mannitol had been administered before referral (0.5 g/kg IV). Neurological examination findings at the time of presentation approximately 6 hours after ROSC included a comatose mental status with fixed, dilated pupils, absent vestibulo‐ocular reflex, and absent gag reflex. A modified Glasgow coma scale (MGCS) of 4 was recorded and remained static over 12 hours.

Neurological examination findings were consistent with a neuroanatomical localization of brainstem (3), diffuse forebrain and brainstem (1) or diffuse forebrain and cerebellum (2). Global hypoxic‐ischemic brain injury was suspected in all cases based on the history of documented cardiopulmonary arrest or poor recovery after a suspected anesthetic complication, the onset of neurological deficits following ROSC/anesthesia and the bilateral symmetrical nature of the neurological deficits.

**FIGURE 1:**
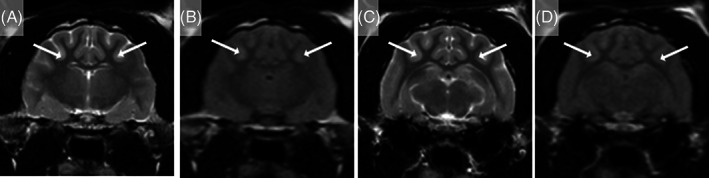
Magnetic resonance images of the head of a 1.5‐year‐old Maine Coon cat that presented for further investigation of cluster seizures after prolonged recovery from general anesthesia for a dental procedure. T2‐weighted (A and C) and T2‐weighted FLAIR (B and D) transverse images at the level of the interthalamic adhesion (A and B) and medial geniculate nuclei (C and D) show mild, ill‐defined areas of hyperintensity of the cerebral gray matter (arrows) of the parietal and occipital lobes secondary to presumed global HIBI. Hyperintensities are reported relative to normal gray matter.

**FIGURE 2:**
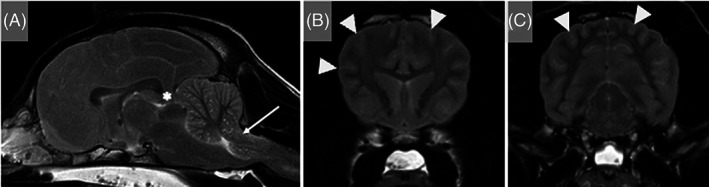
Magnetic resonance images of the head of a 2.5‐year‐old intact male spaniel crossbreed dog that presented after cardiopulmonary arrest and successful resuscitation at the referring veterinary surgeon's hospital. T2‐weighted sagittal (A) and transverse images at the level of the head of the caudate nuclei (B) and rostral colliculi (C) show a diffusely swollen brain with almost complete obliteration of the cerebral sulci (arrowheads) and ventricular compression. There is diffuse bilateral subtle to moderate loss of gray and white matter differentiation. These changes are more severe dorsally within the parietal and occipital lobes. There is caudal transtentorial herniation (asterisk) with compression and caudal displacement of the rostral cerebellum. Additionally, the cerebellum is protruding into the foramen magnum (arrow) with severe compression of the underlying medulla oblongata. The cervical spinal cord is swollen with obliteration of the surrounding cerebrospinal fluid signal. The caudal aspect of the nasal cavity, nasopharynx, caudal aspect of the oral cavity and frontal sinuses contains T2W hyperintense material (likely compatible with regurgitation of gastric contents). Hyperintensities are reported relative to normal gray matter.

**FIGURE 3:**
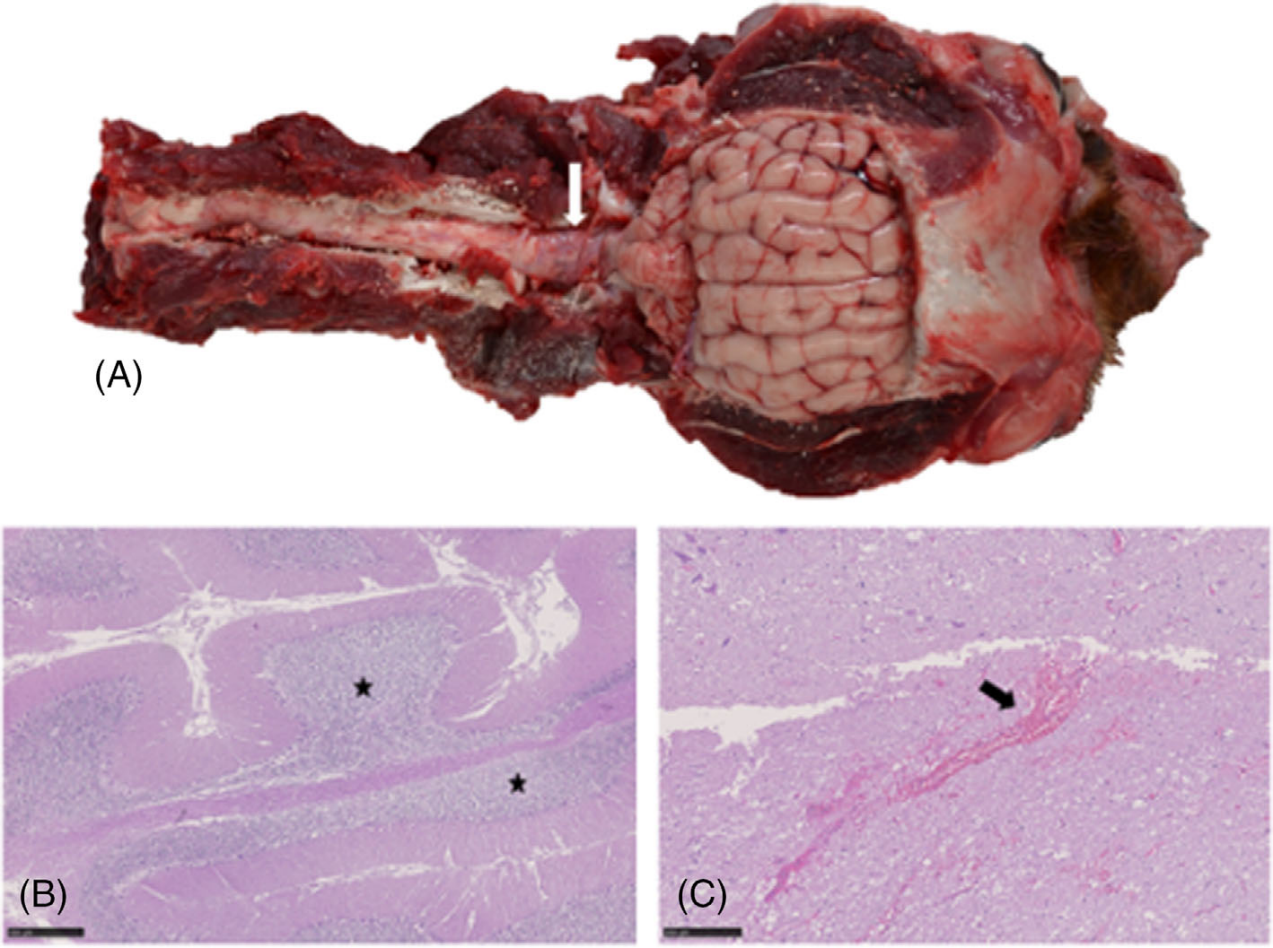
Gross pathology and histopathology of the brain of a 2.5‐year‐old intact male spaniel crossbreed dog that was euthanized after in‐hospital cardiopulmonary arrest. (A) Head and proximal cervical spinal cord with the caudodorsal calvarium and dorsal vertebral column removed. The cerebellum is positioned caudally within the skull (associated with in situ foramen magnum herniation) and the brainstem and proximal cervical spinal cord are reddened (arrow). (B) Cerebellum, Hematoxylin & eosin (H&E). Mild cerebellar edema in the granular cell layer (asterisk). Scale bar, 500 μm. (C) Proximal cervical spinal cord, H&E. Multifocal areas of acute hemorrhage expanding the gray and white matter (arrow). Scale bar, 250 μm.

In the Maine Coon cat, additional diagnostic investigations included magnetic resonance imaging (MRI) of the head performed 24 hours after presentation. This procedure identified mild bilaterally symmetrical, ill‐defined T2‐weighted (T2W) and T2W fluid‐attenuated inversion recovery (FLAIR) hyperintensity of the cortical gray matter of the parietal and occipital lobes (Figure 1). In the BSH cat, electroencephalography (EEG) was performed 6 hours post‐ROSC and identified continuous low‐voltage background activity of approximately 15 μV, 10 Hz. The spaniel crossbreed dog that presented comatose underwent assessment of somatosensory evoked potentials after left and right sciatic nerve stimulation, which showed no detectable response. The EEG was characterized by suppressed background activity (<10 μV). The MRI of the head (T2W and T2 gradient echo sequences only) identified generalized T2W hyperintensity of the cerebral gray matter that appeared diffusely swollen with loss of distinction between the gray and white matter, most apparent in the parietal and occipital cortex. Obliteration of the cerebral sulci, compression of all ventricles, and caudal transtentorial and foramen magnum herniation with severe brainstem compression were observed (Figure 2). These changes were considered most likely to represent severe cytotoxic edema secondary to GHIBI. Given the severity of the clinical presentation, the MRI features and the electrodiagnostic evaluations, the dog was euthanized. Necropsy 24 hours after euthanasia identified diffuse swelling of the brain with compression of the cerebellum, brainstem, and proximal cervical spinal cord (Figure 3A). Histopathological examination disclosed mild cerebellar edema in the granular cell layer and multifocal areas of acute hemorrhage within the proximal cervical spinal cord (Figure 3B,C).

Treatment in the remaining 7 cases consisted of supportive nursing care including regular turning and assistance to eat and drink (7), physiotherapy (6), antiepileptic medication (3) (phenobarbital [1], levetiracetam [2]), oxygen therapy (1), placement of a nasogastric tube to facilitate provision of enteral nutrition (1) and a packed red blood cell transfusion in the dog with severe hemorrhagic gastroenteritis (1). Improvements in mentation and ambulation were first documented in all 7 animals within 48‐72 hours of hospitalization, and further improvements typically were documented every 48 hours thereafter. Serial assessment of the MGCS was recorded in 2 dogs and both cats. On presentation, a MGCS of 7 was documented in the Shih Tsu, with progressive improvements over the next 24 hours to a MGCS of 13. It then remained stable at MGCS 13 for an additional 24 hours when recording was discontinued. In the small crossbreed dog with hemorrhagic gastroenteritis, a MGCS of 12 was documented on presentation, which increased to MGCS 16 over the next 9 hours, where it remained stable for the next 72 hours when recording was discontinued. The Maine Coon cat had an initial MGCS of 11, increasing to 17 after 33 hours, where it remained stable until recording was discontinued at 72 hours. Finally, the BSH had a MGCS of 4 documented 5 hours after cardiopulmonary arrest; MGCS increased to 12 after 21 hours, and 13 after an additional 12 hours when recording was discontinued.

Median duration of hospitalization was 7 days (range, 2‐19 days). Short‐term outcome was documented as survival at 72 hours in 7 cases and non‐survival in 1 case. Re‐evaluation in the 7 animals that survived to hospital discharge was performed after a median of 67 days (range, 12‐180 days). Re‐evaluation identified persistent neurological deficits in 2 dogs (Shih Tzu and small crossbreed) and 1 cat (Maine Coon), including decreased to absent menace response bilaterally (3), impaired vision (2), absent vision (1), ambulatory tetraparesis (1) and mild generalized hypermetria (1). Three dogs (CKCS, Labrador retriever and Bichon Frise) and 1 cat (BSH) recovered fully with no neurological deficits detected on re‐examination 4‐12 weeks post‐GHIBI. Therefore, 1 cat (Maine Coon) had a long‐term outcome score of 1, 2 dogs had a score of 2 whereas the remaining 4 animals had a score of 3. Antiepileptic medication was continued in the 3 animals observed to have seizures during hospitalization; phenobarbital was continued for 8 months in the Labrador retriever and then tapered over the subsequent 4 months before discontinuing. At follow‐up 4 years later, no additional seizures had been observed. The CKCS was continuing to receive levetiracetam 3 months after hospital discharge, but no additional seizures had been observed. Levetiracetam was discontinued after 8 weeks in the Maine Coon cat and 15 months post‐GHIBI no further seizures had been observed.

### 3.2 Out‐of‐hospital cardiopulmonary arrest

A 6‐month‐old intact male Weimaraner presented in cardiopulmonary arrest after a road traffic accident and a 7‐month‐old intact male Cocker Spaniel presented apneic with no palpable peripheral pulses secondary to laryngeal obstruction by a foreign body. Both dogs were reported to have been apneic for approximately 2 minutes before arriving at the hospital. The Weimaraner was resuscitated after approximately 10 minutes of cardiopulmonary arrest and the Cocker Spaniel after 22 minutes, including external defibrillation.

**FIGURE 4:**
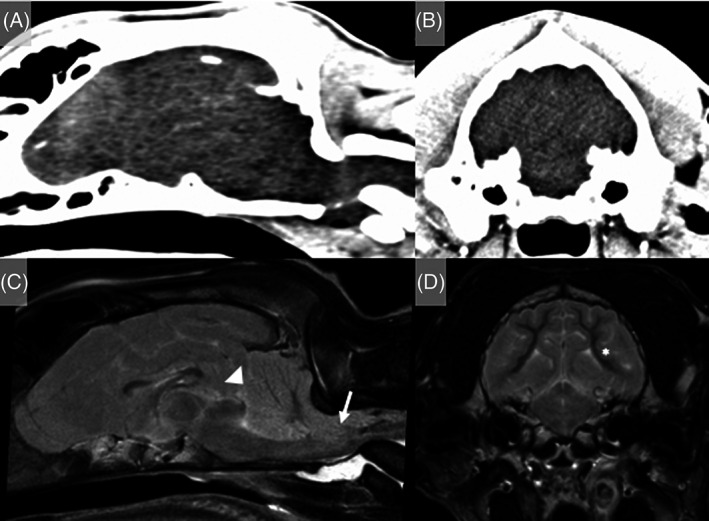
A 6‐month‐old intact male Weimaraner that presented after an out‐of‐hospital cardiopulmonary arrest secondary to trauma. (A and B) CT reconstruction of the head showed no evidence of head trauma. (C and D) MRI of the head (T2W sagittal [C] and transverse image at the level of the rostral colliculi [D]) identified diffuse loss of differentiation between cerebral gray and white matter, diffuse cortical hyperintensity on T2W with swelling of the cortical gray matter (asterisk), loss of cerebral sulci, and severe foramen magnum (arrow) and caudal transtentorial herniation (arrowhead) with secondary brainstem compression. These changes were consistent with a global HIBI and euthanasia was elected given the severity of the clinical presentation and MRI findings. Necropsy was not performed. Hyperintensities are reported relative to normal gray matter.

After ROSC, the Weimaraner was comatose with fixed dilated pupils, absent vestibulo‐ocular reflex, absent gag reflex, absent palpebral reflex and absent corneal reflex (Video S3). Neuro‐localization was diffuse brainstem. The dog was intubated and manually ventilated. Mannitol (1 g/kg IV) was administered but no change in neurological status was observed. Computed tomography (CT) of the thorax and abdomen identified changes consistent with pulmonary contusions as well as a small volume of peritoneal fluid, suspected to be hemorrhage. The CT of the head showed no evidence of head trauma (Figure 4A,B). The MRI of the head (T2W, T2W FLAIR, and T2 gradient echo sequences only; Figure 4C,D) disclosed diffuse cerebral cortical swelling with hyperintensity on T2W images, loss of differentiation between cerebral gray and white matter, loss of cerebral sulci and severe foramen magnum and caudal transtentorial herniation with secondary brainstem compression. These changes were considered consistent with extensive cytotoxic edema secondary to GHIBI. Traumatic brain injury was considered less likely because of the severe, diffuse nature of the identified changes and the absence of skull fractures and hemorrhage. Euthanasia was elected given the severity of the clinical presentation and MRI findings. Necropsy was not performed.

**FIGURE 5:**
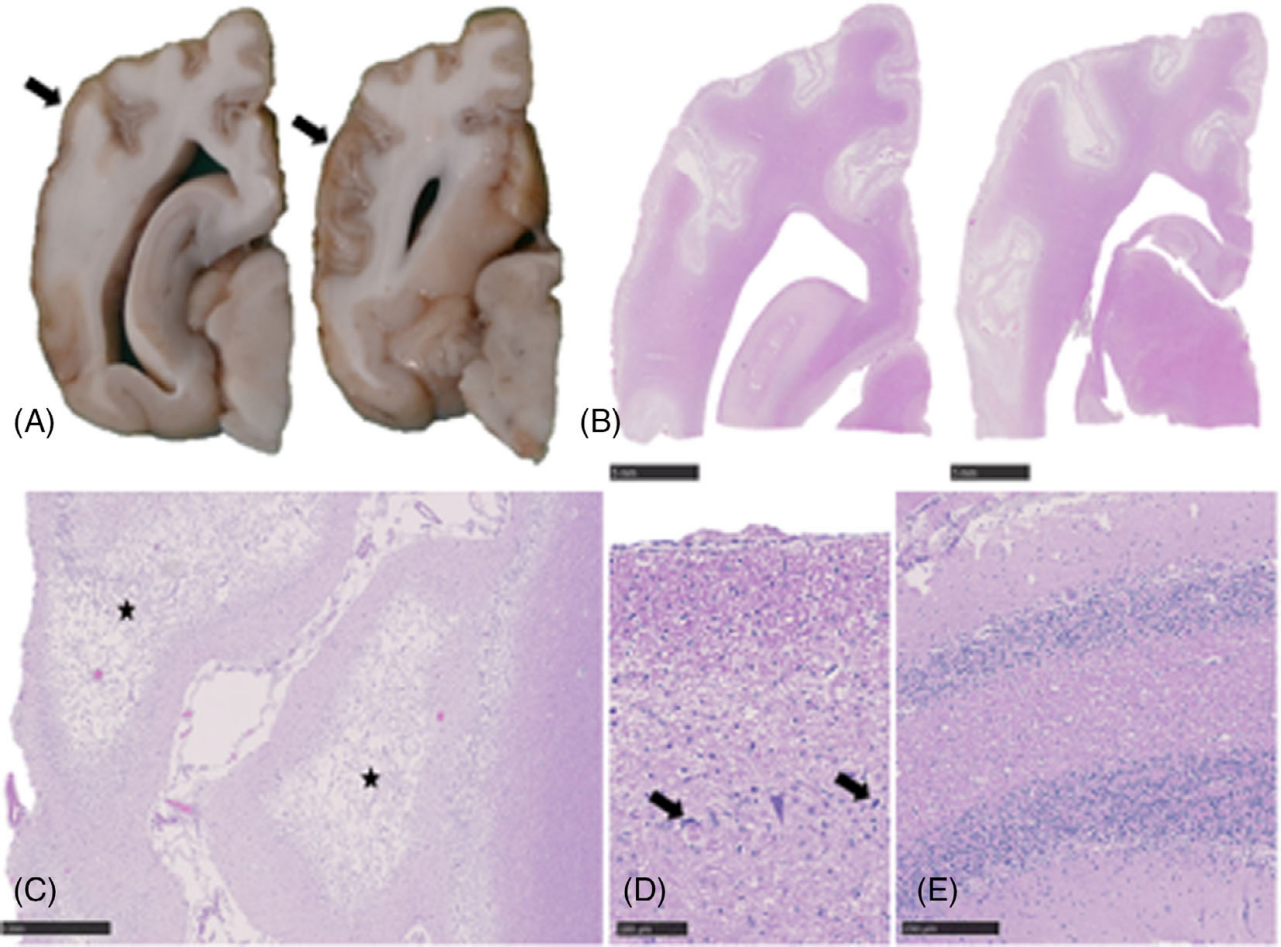
Gross pathology and histopathology of the brain of a 7‐month‐old intact male Cocker Spaniel with asphyxiation secondary to laryngeal obstruction. (A) Cerebrum, formalin fixed serial sections. Severe thinning and discoloration of the parietal and temporal cerebral cortex (arrows). (B) Cerebrum, H&E sections corresponding to (A). Diffuse pallor of the cortical gray matter with areas of neuropil loss. Scale bars, 5 mm. (C) Occipital cortex, H&E. Severe cortical spongiosis and necrosis (asterisk) with neovascularization. Scale bar, 1 mm. (D) Hippocampus, CA1 region, H&E. Severe neuronal degeneration (arrow) and loss. Scale bar, 100 μm. (E) Cerebellar folia, H&E. Marked Purkinje cell loss. Scale bar, 250 μm.

An initial neurological examination of the Cocker Spaniel 3 hours after ROSC showed marked obtundation and disorientation, nonambulatory tetraparesis, absent menace response, and absent vision bilaterally (Video S4). Neuro‐localization was diffuse forebrain and brainstem. Treatment consisted of supportive nursing care, sedation to manage severe disorientation and vocalization (dexmedetomidine and midazolam constant rate infusions), oxygen therapy, supported feeding and physiotherapy. Electroencephalography was performed 2 days post‐resuscitation and identified no evidence of epileptiform activity. Discontinuous low‐voltage background activity of approximately 15 μV was observed. A MGCS of 14 was recorded 6 hours after ROSC and remained stable on repeat assessments over the next 96 hours when recording was discontinued. Very slight improvements in mentation and gait were recorded during hospitalization. The episodic vocalization decreased in frequency over 14 days and the dog regained independent ambulation 17 days post‐ROSC. The dog was discharged for continued rehabilitation at home 25 days post‐ROSC. On re‐examination 18 days after hospital discharge, the dog remained severely disorientated and ambulatory tetraparetic, with hypermetria and vestibular ataxia, circling to the left, resting horizontal nystagmus (fast phase to the right) and absent menace response bilaterally. The dog was euthanized 58 days after presentation because of concerns over quality of life and the severity of the persistent neurological deficits. The long‐term outcome score was 0. Necropsy identified diffuse severe atrophy of the cerebral and cerebellar cortical gray matter, cerebral dorsal flattening, and loss of distinction between the sulci and gyri (Figure 5A). Histopathological examination identified diffuse severe cortical spongiosis and necrosis of the cerebral and cerebellar gray matter with neovascularization, rare ghost neurons and mild to moderate gliosis and microgliosis, respectively. The most severe changes were found in the occipital lobe (Figure 5B,C). Within the hippocampal CA1 region and the cerebellar folia, multifocal moderate neuronal and Purkinje cell loss, respectively, were observed (Figure 5D,E).

## 4 DISCUSSION

Global hypoxic‐ischemic brain injury can result in severe neurological deficits in veterinary patients. We report 8 dogs and 2 cats with GHIBI after documented or suspected cardiopulmonary arrest secondary to a range of causes including general anesthesia, trauma, hemorrhagic gastroenteritis, laryngeal obstruction, and congestive heart failure. A positive outcome (long‐term outcome score of 2 or 3) was documented in 6 of 8 cases with an in‐hospital hypoxic‐ischemic event, likely reflecting prompt recognition of the onset of the hypoxic‐ischemic event and rapid initiation of cardiopulmonary resuscitation. Duration of the hypoxic‐ischemic insult has been shown to have a linear association with neuronal loss and injury severity in a study evaluating perinatal rats.^20^ Furthermore, the cause of the cardiac arrest has been shown to be associated with injury severity; more severe brain injury has been identified after asphyxial cardiac arrest compared to ventricular fibrillation cardiac arrest (VFCA) of the same duration in rats and dogs.^21^, ^22^ In asphyxiation, arterial oxygenation decreases with increasing hypercapnia before the onset of cardiac arrest, whereas oxygenation typically is normal at the onset of VFCA. In a rat model, asphyxial cardiac arrest resulted in more severe early neurologic deficits, increased neuronal loss 7 days post‐arrest and more depleted brain antioxidant reserves.^21^ One dog in this case series presented with asphyxial cardiac arrest secondary to laryngeal obstruction by food material. This dog showed some neurological improvement after ROSC but ultimately was euthanized because of the severity of the persistent neurological deficits.

In our study, 4 dogs experienced cardiopulmonary arrest under general anesthesia. Although not documented, a perianesthetic cardiac or pulmonary arrest under general anesthesia was considered most likely in the Maine Coon cat that experienced prolonged recovery after a dental procedure. That being said, neurological deficits consistent with a GHIBI have been reported in cats after general anesthesia in which no anesthetic complications were documented.^12^ Mortality rates in dogs and cats undergoing anesthesia or sedation have been reported to be 0.24%‐0.9%.^23^, ^24^ However, accurate assessment of the prevalence of anesthesia‐associated complications is challenging. Furthermore, the anesthetics were not administered at our hospital and detailed interrogation of the cause and duration of the anesthetic event was not possible. Post‐anesthesia cortical blindness has been reported in cats, particularly in association with the use of mouth gags during dental procedures.^12^, ^13^, ^25^ Maximal jaw opening has been determined to cause decreases in maxillary artery and cerebral blood flow in some cats.^26^ Details regarding the use of a mouth gag and the duration of anesthesia were not available for the Maine Coon cat in our case series.

In people with GHIBI, the presence of initially severe neurological abnormalities does not preclude acceptable functional outcome.^18^ In all cases in our series, neurological deficits initially were severe and predominantly localized to the forebrain, brainstem or both. Of the 6 cases with positive outcome, consistent neurological improvement was identified with detectable improvement within the first 48‐72 hours after GHIBI onset and further improvement identified typically every 48 hours thereafter. Of the 3 dogs with poor outcome, 2 were euthanized shortly after presentation because of the severity of their neurological deficits consistent with diffuse brainstem involvement and marked brainstem compression secondary to the GHIBI seen on MRI. The final dog ultimately was euthanized because of the severity of ongoing neurological deficits. This dog showed minimal slow improvements in neurological status after ROSC and regained independent ambulation after 17 days. Interestingly, pathological horizontal nystagmus was documented at re‐evaluation 43 days after the hypoxic‐ischemic insult, suggesting ongoing brainstem involvement (with some degree of lateralization). Thus, the presence of neurological deficits consistent with diffuse brainstem involvement, persistence of those deficits, and rate of neurological improvement after GHIBI might provide indications of prognosis. Utilizing grading systems to enable serial quantified recordings of neurological progress might facilitate such assessment, but such scores require validation in veterinary medicine. A neurological deficit scoring system from a post‐cardiopulmonary arrest swine model was adapted in a recent veterinary study, with all 9 dogs alive 1 hour after cardiopulmonary arrest showing improved scores over time.^27^ The MGCS has been validated for use in dogs with traumatic brain injury and might offer a valuable tool for serial evaluation in GHIBI.^28^ A MGCS was recorded infrequently and inconsistently in our cases and hence we have been unable to evaluate its utility in GHIBI. However, serial assessments of the MGCS in the Shih Tsu, small crossbreed dog, Maine Coon cat and BSH cat identified progressive improvements over the initial 9‐33 hours of hospitalization. In contrast, the spaniel with out‐of‐hospital cardiopulmonary arrest that ultimately was euthanized because of the severity of its neurological deficits had a MGCS of 14, which remained static over the next 96 hours. This observation supports the potential value of the MGCS as a quantitative measure of improving neurological status suggestive of a more favorable outcome in GHIBI. Interestingly, the initial MGCS in the spaniel was higher than in the Shih Tsu, crossbreed, Maine Coon cat and BSH cat, which all made favorable recoveries, suggesting that a comparatively less severe initial assessment is not necessarily indicative of a positive long‐term outcome.

Magnetic resonance imaging is routinely used in human GHIBI patients to characterize lesion extent and severity. In particular, identification of diffuse brain injury on MRI, specifically attenuation of the gray and white matter interface, is a negative prognostic indicator and diffusion weighted imaging (DWI) provides prognostic information after cardiopulmonary arrest in people.^29^, ^30^, ^31^, ^32^ Current guidelines used in humans recommend obtaining DWI 2‐5 days after cardiopulmonary arrest to facilitate prediction of neurologic outcome. Evaluated variables include whole brain apparent diffusion coefficient (ADC) quantification, the proportion of brain volume with low ADC or the lowest ADC value in specific sites including the occipital cortex and hippocampus.^31^, ^33^ Furthermore, the absence of DWI abnormalities within 1 week of cardiopulmonary arrest was suggestive of good neurological outcome.^33^ In experimentally induced cardiopulmonary arrest models in dogs^34^ and cats,^35^ DWI and ADC were shown to be useful in monitoring the progression or reversal of ischemic brain injury. In our study, MRI was performed in only 3 cases and DWI was not undertaken. The clinical decision not to proceed with advanced imaging often was multifactorial and based on a high index of suspicion of the underlying diagnosis without the need for MRI confirmation, the concerns of higher general anesthesia risk in patients with a recent cardiopulmonary arrest, cost and low likelihood that MRI would change the ongoing management plan for the patient. Therefore, although MRI can offer information in terms of lesion extent and severity and might support prognostication, the risks and costs also must be considered on a patient‐by‐patient basis and studies to explore alternative tools that do not necessitate general anesthesia would be beneficial.

Electrodiagnostic evaluation is an additional tool used in the evaluation of people after cardiopulmonary arrest. Electroencephalography has been shown to be a useful tool to assess the severity of GHIBI and provide prognostic information.^36^, ^37^ Intermittent EEG is commonly used in clinical practice but continuous EEG monitoring enables assessment of the evolution of brain activity over time.^36^ Patients typically show suppressed background activity patterns (<10 μV) immediately after cardiac arrest, with gradual increases in amplitude and continuity thereafter. Progression toward continuous normal voltage background activity within 24 hours of cardiac arrest has been shown to be associated with good outcome.^38^, ^39^ A poor prognosis was associated with suppressed background activity, a non or poorly responsive EEG pattern (ie, no response to auditory, visual, tactile, or noxious stimuli) and the presence of periodic generalized phenomena.^14^ Isolated discharges on EEG have been shown to have no predictive value but generalized periodic discharges or electrographic seizures are associated with poor neurological outcome.^38^ Global hypoxic‐ischemic brain injury is diffuse and thus decreased montages (e.g., 6 channels) can provide equally reliable results as compared with full montages.^40^, ^41^ In our case series, EEG was performed in only 2 cases. The BSH cat showed continuous low‐voltage background activity 6 hours post‐resuscitation and went on to make a full neurological recovery whereas the Cocker Spaniel with laryngeal obstruction showed discontinuous low‐voltage background activity and failed to make a clinically relevant neurological recovery. Epileptiform discharges were not detected in either, and serial assessments of changes in EEG background activity were not performed. Somatosensory evoked potentials (SSEP) also have been shown to have prognostic utility in people. The bilateral absence of the N20 cortical wave of SSEP 72 hours after ROSC was shown to have high accuracy in predicting poor neurological outcome.^42^ Furthermore, SSEPs are less affected by concurrent sedation but may be disrupted by electrical interference.^33^ Assessment of SSEP was performed in 1 dog (spaniel cross) in our case series 24 hours after the GHIBI. No detectable response was obtained on stimulation of the sciatic nerves, and this dog subsequently was euthanized given the severity of its clinical presentation and MRI findings (severe brainstem compression). The absence of SSEP is potentially a negative prognostic indicator in dogs but future studies are needed to evaluate the utility of both SSEP and EEG in GHIBI in veterinary patients.

Brainstem auditory evoked potentials (BAEP) offer a means to assess the integrity of the central hearing pathways as an indicator of brainstem function. The absence of BAEP has been documented in comatose people and dogs, and there can be loss of all waveforms (consistent with cessation of intracranial circulation) or preservation of waveforms I and II indicating integrity of the cochlear nerve and nucleus.^43^, ^44^ A decrease in waveform amplitude has been recorded in caudal transtentorial herniation,^44^, ^45^ and a study in dogs reported that BAEP were sensitive for the detection of brainstem lesions, while additionally providing information on location and extent of lesions.^46^ Brainstem auditory evoked potentials were not evaluated in the animals in our study but warrant investigation as an additional tool in evaluation and prognostication for GHIBI cases.

Neuron specific enolase (NSE) is released from injured neurons and its plasma concentration correlates with the severity of GHIBI in people.^47^ Other biomarkers of interest include S100‐beta (released from damaged glia) and protein tau (released from damaged axons).^33^ In veterinary medicine, various proteins have been evaluated as potential biomarkers in different diseases, including NSE in dogs with distemper virus encephalitis^48^ and neurofilament light chain in meningoencephalitis of unknown etiology^49^ and cognitive decline.^50^ Serum glial fibrillary acidic protein concentration has been shown to predict outcome in dogs with complete spinal cord injury.^51^ Veterinary studies are needed to evaluate the clinical utility of serum biomarkers in GHIBI.

After resuscitation from out‐of‐hospital cardiac arrest, 80% of people who are admitted to an intensive care unit are comatose and prognostication is needed to guide decision making over whether life support should be continued.^33^, ^52^ In our case series, 2 dogs presented comatose and required mechanical ventilation. Both dogs were euthanized based on the severity of the neurological deficits and the severity of MRI changes, including severe foramen magnum herniation and brainstem compression secondary to GHIBI. The prognosis was deemed to be grave and the owners were counseled on the low likelihood of functional recovery. However, the European Resuscitation Council and European Society of Intensive Care Medicine guidelines recommend delaying neurological prognostication for at least 72 hours after ROSC in people.^2^ To date, no equivalent recommendation exists in veterinary medicine, but consideration should be given to ensuring sufficient time is given for a multimodal approach to prognostication including serial neurological assessments, advanced imaging, serum biomarker evaluation and electrodiagnostic testing. Such a multimodal approach also could support clinical decision making in less severely affected animals to avoid intensive and prolonged efforts at rehabilitation if the ultimate neurological recovery and associated quality of life will not reach a sufficient level.

Our study had some limitations. Importantly the number of cases available for inclusion was small and the retrospective nature of the study inherently was associated with variations in clinical record detail and clinical management decisions. Detailed information on the anesthetic monitoring and cardiopulmonary resuscitation performed at the referring veterinary practices was not recorded in the available clinical notes. Confirmation of GHIBI by histopathological evaluation was performed in only 2 dogs, with advanced imaging of the brain undertaken in 2 dogs and 1 cat. Concurrent metabolic or cardiovascular derangements might have contributed to the encephalopathy in some cases (e.g., the dogs with pyometra and hemorrhagic gastroenteritis) and complicated the potential for accurate prognostication by exacerbating clinical signs or delaying the rate or extent of recovery. A further limitation was that follow‐up times were limited and the timing of reassessments varied among patients. A more detailed and consistent assessment of recovery at specific time points after hospital discharge would facilitate accurate evaluation of long‐term outcome. Cases with more severe GHIBI might have died before referral or euthanasia might have been elected before referral. Furthermore, all included cases were referred to a veterinary specialist and hence are unlikely to represent the full spectrum of GHIBI in dogs and cats.

In conclusion, GHIBI in dogs and cats is associated with a range of etiologies. Despite severe neurological deficits at onset, long‐term positive outcome is possible. The duration of the hypoxic‐ischemic insult and the rate of subsequent neurological improvement might provide indications of the likelihood of functional recovery in dogs and cats with GHIBI, with longer insults (particularly where resuscitation is delayed by out‐of‐hospital arrest) and slow neurological improvement after ROSC suggestive of a worse long‐term outcome, as well as diffuse brainstem involvement. Studies to develop EEG prognostic indicators and plasma biomarkers should support clinical decision making and prognostication in veterinary GHIBI patients.

## CONFLICT OF INTEREST DECLARATION

Authors declare no conflict of interest.

## OFF‐LABEL ANTIMICROBIAL DECLARATION

Authors declare no off‐label use of antimicrobials.

## INSTITUTIONAL ANIMAL CARE AND USE COMMITTEE (IACUC) OR OTHER APPROVAL DECLARATION

Authors declare no IACUC or other approval was needed.

## HUMAN ETHICS APPROVAL DECLARATION

Authors declare human ethics approval was not needed for this study.

## Supporting information


**Video S1:** A 6‐year‐old female neutered Shih Tsu presented 12 hours after cardiopulmonary arrest during an exploratory laparotomy to treat pyometra. The dog was stuporous with absent menace response bilaterally, absent vision, miotic pupils, and a markedly decreased vestibulo‐ocular reflex. The dog showed progressive improvements with supportive care and at hospital discharge 15 days post‐GHIBI was ambulatory, with mild obtundation and disorientation, absent menace response and absent vision bilaterally.

**Video S2:** A 5‐year‐old female neutered cross breed dog presented after cardiopulmonary arrest at the referring veterinary hospital secondary to hypovolemia associated with severe hemorrhagic gastroenteritis. The dog was severely obtunded, and non‐ambulatory tetraparetic with absent menace response and absent vision bilaterally. The dog showed progressive improvements with supportive care and at the time of hospital discharge 19 days post‐admission was ambulatory with mild hypermetria, an intention tremor, absent menace response, and absent vision bilaterally. Serial reassessments documented a decreased menace response in the left eye and an ability to navigate unfamiliar environments if well lit.

**Video S3:** A 6‐month‐old intact male Weimaraner presented to the referring veterinary surgeon in cardiopulmonary arrest after a road traffic accident and was resuscitated after approximately 10 minutes. The dog was then transferred to our hospital intubated and manually ventilated. The dog was comatose with fixed dilated pupils, absent vestibulo‐ocular reflex, absent gag reflex, absent palpebral reflex, and absent corneal reflex.

**Video S4:** A 7‐month‐old intact male Cocker Spaniel presented apneic with no palpable peripheral pulses secondary to laryngeal obstruction by a foreign body. Return of spontaneous circulation was documented after 22 minutes of resuscitation. Neurological examination 3 hours after presentation showed marked obtundation and disorientation, non‐ambulatory tetraparesis and absent menace response bilaterally with absent vision bilaterally. Very slight improvements in mentation and gait were recorded during 25 days of hospitalization, after which the dog was discharged for continued rehabilitation at home. On re‐examination 6 weeks after the GHIBI, the dog remained severely disorientated, ambulatory tetraparetic with hypermetria and vestibular ataxia, circling to the left, resting horizontal nystagmus (fast phase to the right) and absent menace response bilaterally.
